# The Geriatric Nutritional Risk Index Predicts Survival in Elderly Esophageal Squamous Cell Carcinoma Patients with Radiotherapy

**DOI:** 10.1371/journal.pone.0155903

**Published:** 2016-05-19

**Authors:** Yacong Bo, Kunlun Wang, Yang Liu, Jie You, Han Cui, Yiwei Zhu, Quanjun Lu, Ling Yuan

**Affiliations:** 1 Department of Nutrition and Food Hygiene, College of Public Health, Zhengzhou University, Zhengzhou, China; 2 Department of radiotherapy, Henan Tumor Hospital, Affiliated Tumor Hospital of Zhengzhou University, Zhengzhou, China; Taipei Medical University, TAIWAN

## Abstract

The impact of nutritional status on survival among elderly esophageal squamous cell carcinoma (ESCC) patients undergoing radiotherapy is unclear. In this study, we aimed at validating the performance of the geriatric nutritional risk index (GNRI) in predicting overall survival time in elderly ESCC patients with radiotherapy. A retrospective cohort study was conducted on 239 ESCC patients aged 60 and over admitted consecutively from January 2008 to November 2014 in the Department of Radiotherapy, Henan Tumor Hospital (Affiliated Tumor Hospital of Zhengzhou University), Zhengzhou, Henan, China. All patients were subjected to nutritional screening using GNRI, and were followed for the occurrence of lymphatic node metastasis, radiation complication and mortality. The Kaplan–Meier method with Log-rank test was used to estimate survival curves. Univariable Cox regression analysis was used to identify variables associated with overall survival time. Among the 239 patients, 184 patients (76.9%) took no nutritional risk, 32 patients (13.4%) took moderate risk of malnutrition, and 23 patients (9.7%) took a high risk of malnutrition. Univariable Cox regression showed that both high nutritional risk group and moderate nutritional risk group were significantly less likely to survive than no nutritional risk patients (hazard ratio (HR) = 1.688, 95% confidence interval (CI) = 1.019–2.798 for moderate risk group, and HR = 2.699, 95% CI = 1.512–4.819 for high risk group, respectively). The GNRI is an independent prognostic factor for overall survival time in elderly ESCC patients with radiotherapy. A GNRI ≤98 can be suggested as an indicator of surviving less.

## Introduction

Esophageal cancer ranks the eighth leading cause of cancer-related deaths and the tenth most common malignancy worldwide.[1] And China is the country with top prevalence and mortality of esophageal cancer, especially esophageal squamous cell carcinoma (ESCC). Malnutrition commonly observed in esophageal cancer patients,[2] and the presence of malnutrition is associated with poor clinical outcomes: impairing quality of life, performance status, immune functions, muscle function, and even survival in esophageal cancer patients. [3] It has been recognized that age is an independent predictor of poor clinical outcome and nutritional disorders,[4, 5] but it is frequently unrecognized.

The GNRI, a screening index of nutrition-related risk, is an objective and simple nutritional assessment option determined by only serum albumin and body weight. This index was established by Bouillanne et al.[6] It has been proposed for the evaluation of at-risk elderly hospital patients,[7–11] chronic obstructive pulmonary disease,[12] hemodialysis patients,[13–17] and cardiovascular patients.[18–20] To date, no long-term population-based cohort studies have estimated the association between the GNRI and the survival of ESCC patients. Thus, the present study aimed to investigate whether the GNRI is a reliable predictor of the survival in elderly ESCC patients who undergone radiotherapy.

## Methods and Material

### Participants

The participants should meet all the following criteria: (1) aged 60 years old or older; (2) pathological diagnosis as ESCC; (3) conscious, able to stand and answer questions; (4) received radiotherapy only; The exclusion criteria for patients were as follows: (1) a pathological diagnosis of esophageal cancer other than ESCC; (2) aged less than 60 years old; (3) presence of malnutrition that resulted from other disease; (4) received surgery or chemotherapy other than radiotherapy.

The 239 ESCC patients aged 60 and over admitted consecutively from January 2008 to November 2014 in the Department of Radiotherapy, Henan Tumor Hospital(Affiliated Tumor Hospital of Zhengzhou University), Zhengzhou, Henan, China were selected. The project was approved by the ethical committee Zhengzhou University. And all the participants signed the informed consent.

### Nutritional assessment by GNRI

The data of weight, height, and serum albumin of the subjects were collected. Nutrition-related complications were assessed according to GNRI.[6, 21] The GNRI, combining two nutritional indicators: albumin and actual weight compared with ideal body weight, was developed by modifying the nutritional risk index for elderly patients.[6, 7, 11] The GNRI formula is as follows:

GNRI = [1.487× serum albumin (g/L) + [41.7× present/usual weight (kg)]

The participants were classified according to the following cut-offs: high risk, <92; moderate risk, 92 to 98; no risk, >98[21].

Similarly to previous study,[7, 11, 22–24] we utilized the modified categories of GNRI: severe risk (GNRI < 92) and moderate risk (GNRI 92–98) categories were included into one single category, as both groups have been demonstrated to present a high risk of complications.[6]

### Follow-up

The primary study outcome was overall survival time, and the second outcomes of follow-up evaluations were lymph node metastasis and radiation complication. Follow-up evaluations were performed every 3 months for the first year, every 6 months for the second year, and yearly thereafter. Follow-up was performed until patient death, or until October 2015, which was the cut-off date for this study.

### Statistical Analysis

The Analysis of variance (ANOVA) was used to examine the differences of continuous variable (age), and the chi-square test was used to explore the difference of categorical variables (including sex, differentiation, tumor location, tumor stage, dose radiotherapy, lymph node metastasis, and radiation complication). The Kaplan–Meier method with Log-rank test was used to estimate survival curves. Univariable Cox regression analysis was used to identify variables associated with overall survival time. Variables with a *P* <0.05 on univariable analysis were further assessed with a multivariable Cox regression model. SPSS 21 software (IBM Corp, Armonk, NY) was used for the statistical analysis. The level of significance was established as a two-sided *P* value 0.05.

## Results

### Patient characteristics

One hundred and fifty males and 89 females, whose ages ranged from 60 to 88 years (mean age of 67.9±5.9 years at diagnosis), were included in our study. According to the TNM categories, 22 patients (7%) were classified as stage I, 138(43.9%) were classified as stage II, 54(17.2%) were classified as stage III, and 25(8%) were classified as stage IV. 71(29.7%) patients received radiation doses of ≤50Gy and 168(70.3%) received radiation doses of >50Gy. 82(34.3%) patients had metastatic lymph nodes, and 129(53.9%) patients had complication of radiotherapy.

By October, 2015, 226 participants had been followed up.13 patients were lost to follow-up, the follow-up rate was 94.6%. The mean and median survival times were 40.4 months and 33 months, respectively. The cumulative survival probabilities at 1, 3, and 5 years of the 239 participants were 72.6%, 48.9%, and 22.7%, respectively.

As shown in Table 1. 184 patients (76.9%) took no nutritional risk, 32 patients (13.4%) took moderate risk of malnutrition, and 23 patients (9.7%) took a high risk of malnutrition. There were no statistically significant differences in the rate of sex, differentiation, tumor location, tumor stage, dose radiotherapy, lymph node metastasis, diabetes, white blood cell count(WBC), neutrophils, and lymphocyte among different GNRI categories(*P*>0.05). However, the age, weight loss, radiation complication, and serum albumin differentiate among different GNRI categories (*P*<0.05).

**Table 1:** Characteristics of the participants according to geriatric nutritional risk index categories.

Variable	Total(n = 239)	Geriatric Nutritional Risk Index (GNRI)			*P*
		High risk <92 (N = 23)	Moderate risk 92–98 (N = 32)	No risk >98 (N = 184)	
Age at diagnosis (years)	239	71.3±6.1	65.8±4.6	67.8±5.9	0.003
Sex					0.505
Male	150	17(11.3%)	20(13.3%)	113(75.3%)	
Female	89	6(6.7%)	12(13.5%)	71(79.8%)	
Differentiation					0.419
Well	35	5(14.3%)	6(17.1%)	24(68.6%)	
Moderate	51	2(3.9%)	8(15.7%)	41(80.4%)	
Poor	153	16(10.5%)	18(11.8%)	119(77.8%)	
Tumor location					0.117
Upper esophagus	68	3(4.4%)	9(13.2%)	56(82.4%)	
Middle esophagus	143	14(9.8%)	21(14.7%)	108(75.5%)	
Lower esophagus	28	6(21.4%)	2(7.1%)	20(71.4%)	
Tumor stage					0.886
I	22	1(4.5%)	2(9.1%)	19(86.4%)	
II	138	12(8.7%)	19(13.8)	107(77.5%)	
III	54	7(13%)	7(13%)	40(74%)	
IV	25	3(12%)	4(16%)	18(72%)	
Dose radiotherapy (Gy)					0.436
≤50	71	9(12.7%)	11(15.5%)	51(71.8%)	
>50	168	14(8.3%)	21(12.5%)	133(79.2%)	
Lymph node metastasis					0.349
Yes	82	11(13.4%)	10(12.2%)	61(74.4%%)	
No	157	12(7.6%)	22(14.1%)	123(78.3%%)	
Radiation complication					
Radiation esophagitis					0.000
No	115	9(7.8%)	11(9.6%)	95(82.6%)	
Grade 1–2	82	3(3.7%)	11(13.4%)	68(82.9%)	
Grade 3–4	42	11(26.2%)	10(23.8%)	21(50.5%)	
Bone marrow suppression					0.000
No	118	11(8.5%)	10(9.3%)	97(82.2%)	
Grade 1–2	90	4(4.4%)	15(16.7%)	71(78.9%)	
Grade 3–4	31	9(29.0%)	6(19.4%)	16(51.6%)	
Diabetes					0.499
Yes	21	3(9.2%)	14(12.8%)	14(78.0%)	
No	218	20(14.3%)	28(19.0%)	170(66.7%)	
Albumin	239	34.5±10.66	36.8±1.66	42.7±2.69	0.000
WBC(×10^9^/L)	239	6.48±2.37	7.03±2.57	6.58±1.26	0.568
Neutrophils(×10^9^/L)	239	4.02±2.05	4.37±2.22	3.93±1.67	0.429
Lymphocyte(×10^9^/L)	239	1.67±0.69	1.83±0.49	1.94±0.83	0.270
Weight loss(%)	239	19.3±19.7	2.19±4.20	0.58±2.22	0.000

This ordered logistic regression analysis identified only independent predictor for nutritional index categories-weight loss (Table 2).

**Table 2:** Results of Ordered Logistic Regression Analysis for Nutritional Risk Index Categories by Forward Selection.

Variable	Coefficient	Standard Error	Wald	*P*	95%*CI*	
					Lower	Upper
Constant 1	-7.305	2.466	8.772	0.003	-12.139	-2.471
Constant 2	-5.748	2.442	5.540	0.019	-10.535	-0.962
Age at diagnosis(year)	-0.038	0.021	3.229	0.072	-0.080	0.003
Albumin	-0.039	0.047	0.685	0.408	-0.132	0.053
Weight loss(%)	18.965	3.916	23.460	0.000	11.291	26.639
Bone marrow suppression(Grade1-2)	-0.235	0.897	0.069	0.793	-1.993	1.522
Bone marrow suppression(Grade3-4)	-0.407	0.830	0.241	0.623	-2.034	1.219
Radiation esophagitis(Grade1-2)	0.608	0.852	0.509	0.476	-1.062	2.278
Radiation esophagitis(Grade3-4)	-0.559	0.835	0.447	0.504	-2.196	1.079

### Univariate analysis for the survival of elderly ESCC patients

**Fig 1:**
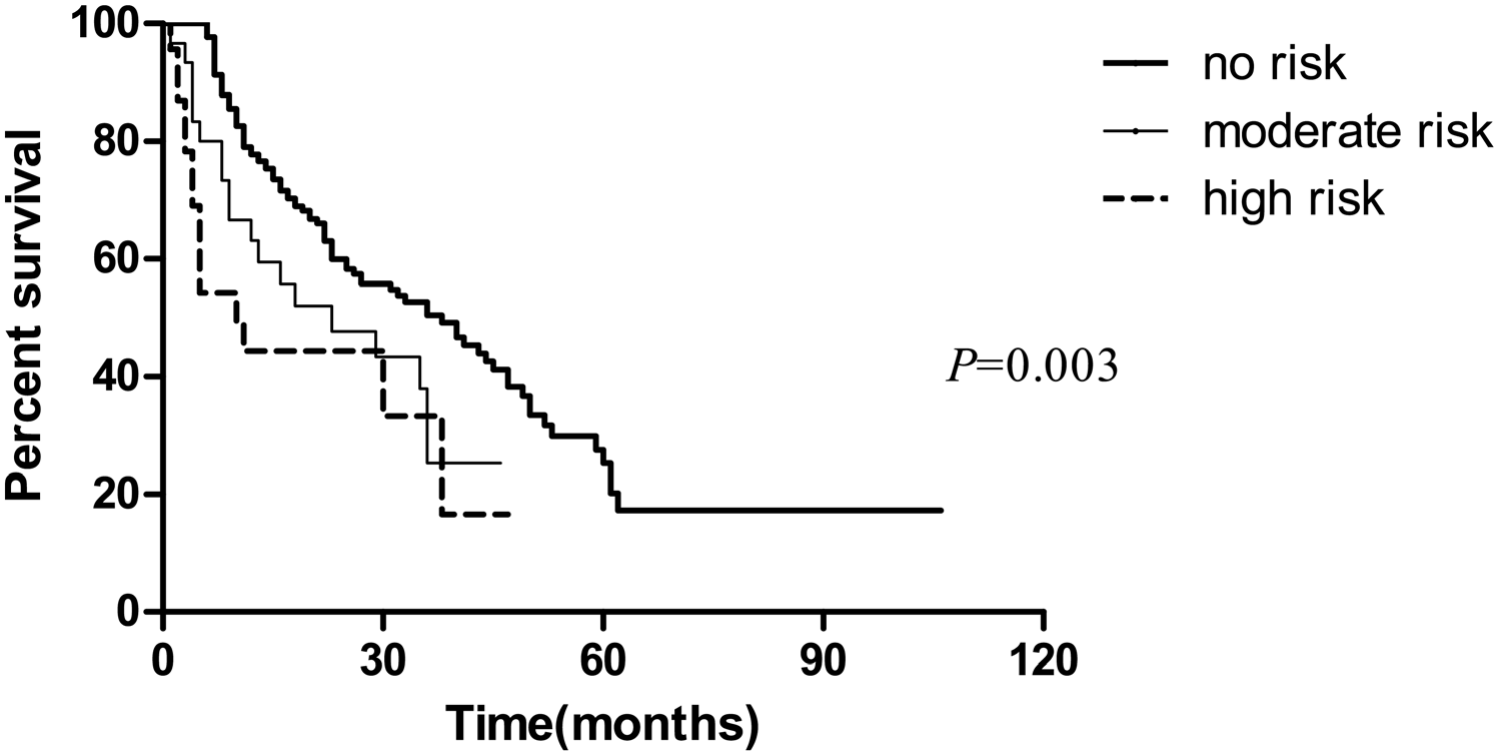
Kaplan–Meier survival curves showing patient overall survival stratified by GNRI categories (high risk, <92; moderate risk, 92 to 98; no risk, >98.

Univariate analysis was performed to identify the factors predicting the survival of elderly ESCC patients who undergone radiotherapy. Survival curves were significantly stratified by GNRI categories (Fig 1). The median survival of the non-nutritional risk group was twice longer than that of the patients with high-nutritional risk (38 months vs. 20 months). Compared with the patients taking no nutritional risk, the risk of death increased by 66.7% (HR = 1.667, 95%CI: 1.011–2.750) for the patients with moderate risk and 135% (HR = 2.350, 95%: 1.331–4.150) for the patients with high risk, respectively. Compared with those patients who had no lymph node metastasis, the patients with lymph node metastasis had higher risk of death (HR = 2.086, 95%CI: 1.464–2.972). Patients with radiation complications had significantly poorer outcomes compared with those patients having no radiation complications (HR = 1.737, 95%CI: 1.213–2.487). We also analyzed whether the poor survival of elderly ESCC patients was caused by other underlying factors, including age, sex, differentiation, tumor location, tumor stage, and the dose of radiotherapy. No significant differences between these subgroups were found (Table 3).

**Table 3:** Univariate analysis of factors associated with overall survival.

Variable	No.	Median survival time (month)	HR	95% CI	*P* Value
Age at diagnosis					
<70(reference)	151	36	1		
≥70	88	27	1.144	0.794–1.648	0.470
Sex					
Male(reference)	150	27	1		
Female	89	40	0.833	0.697–1.002	0.052
Differentiation					
Well(reference)	35	40	1		
Moderate	51	47	0.624	0.348–1.119	0.114
Poor	153	30	0.762	0.465–1.249	0.218
Tumour location					
Upper(reference)	68	29	1		
Middle	143	36	1.052	0.566–1.957	0.872
Lower	28	27	1.022	0.578–1.809	0.940
Tumour stage					
I-II(reference)	160	40	1		
III-IV	79	27	1.145	0.787–1.665	0.480
Dose radiotherapy (Gy)					
≤50(reference)	71	30	1		
>50	168	36	0.985	0.675–1.436	0.936
Lymph node metastasis					
No(reference)	157	40	1		
Yes	82	15	2.086	1.464–2.972	0.000
Radiation complication					
No(reference)	110	40	1		
Yes	129	22	1.737	1.213–2.487	0.003
Diabetes					
No(reference)	218	35	1		
Yes	21	22	1.333	0.735	2.418
GNRI					
>98(reference)	184	38	1		
92–98	32	23	1.667	1.011–2.750	0.045
<92	23	10	2.350	1.331–4.150	0.003

Abbreviations: HR: hazard ratio; CI: confidence intervial

### The multivariable analysis with Cox regression

In order to exclude the impact of some confounders on overall survival time, variables with a *P*<0.05 on univariable analysis were further assessed with a multivariable Cox regression model. We found that GNRI, lymph node metastasis, and radiation complications were independent predictors of overall survival time. Compared with the non-nutritional risk group, the risk of death increased by 68.8% (HR = 1.688, 95%CI: 1.019–2.798) for moderate risk group and 169.9% (HR = 2.699, 95%CI: 1.512–4.819) for high risk group, respectively. (Table 4)

**Table 4:** Multivariable Cox Regression (Outcome Death).

Variable	β	SE	Wald	*P*	HR	95%CI
GNRI						
>98(reference)			13.685	0.001		
92–98	0.524	0.258	4.13	0.042	1.688	1.019–2.798
<92	0.993	0.296	11.273	0.001	2.699	1.512–4.819
Lymph node metastasis (Yes vs. No)	0.691	0.184	14.06	0.000	1.996	1.391–2.864
Radiation complication (Yes vs. No)	0.513	0.188	7.456	0.006	1.671	1.156–2.415

Abbreviations: GNRI: Geriatric Nutritional Risk Index; HR: hazard ratio; CI: Confidence Interval

## Discussion

It is reported that clinical malnutrition can make it more difficult to recovery from disease, trauma and surgery. Malnutrition is associated with increased morbidity and mortality in both chronic and acute conditions. As a result, the duration of hospital treatment is significantly longer in malnourished patients. Hence, the costs is higher.[4] Elderly patients frequently have compromised nutritional status and are also vulnerable to cancer-related deaths.

To our knowledge, this is the first study to evaluate the impact of GNRI on the survival of elderly ESCC patients treated with radiotherapy. We found that patients with malnutrition before radiotherapy take significantly higher risk of mortality. These results suggest that the early nutrition evaluation using GNRI is not only helpful to predict mortality in patients with ESCC, but also an important basis for individualized nutrition and health care, which may be beneficial to these patients.

Due to the diversity of influencing factors of nutritional status in patients with cancer, it is difficult to study the nutritional status of cancer patients.[25] Therefore, whether malnutrition is a potential predictor of surviving less is unclear. In the present study, we performed multivariate analyses adjusting for potential covariates and found that malnutrition is an independent risk factor for death. Compared with the non-nutritional risk group, the risk of death had increased by 68.8% for moderate risk group and 169.9% for high risk group, respectively.

The main advantages of the GNRI are the lower bias associated with past unintentional weight loss investigations and less requirements of participation from participants.[21] Previous studies have demonstrated its prognostic role in rehabilitative, sub-acute, and long-term care settings.[6, 21] However, there is no study to explore the association between GNRI and ESCC survival. To date, there was only a small study that showed an association between GNRI and the short-term postoperative respiratory complications in patients with esophageal cancer undergoing esophagectomy and gastric tube reconstruction.[26] In the present study, the outcomes were overall survival time, which represents a long-term prognosis of ESCC.

Cereda E et al. found that GNRI is a good predictor of length of staying and in-hospital weight loss in elderly patients.[11] Matsumura et al. suggested that GNRI had a more close relation with exercise tolerance and might be a useful nutritional assessment scale for elderly patients with chronic obstructive pulmonary disease (COPD).[12] Besides, GNRI is a higher prognostic value for predicting nutritional-related complications in hospitalized elderly patients.[7] In a prospective cohort study of 332 patients, GNRI is a strong predictor of overall mortality in hemodialysis patients.[27] At the present, we found that GNRI is an independent predictor of overall survival time in elderly ESCC patients, which adds new evidence for the relationship between GNRI and outcomes of elderly patients.

This study had several limitations. Firstly, this study only included elderly ESCC patients who undergone radiotherapy, and the results may not represent a general population of older ESCC patients undergoing other therapy methods(such as esophagectomy and chemotherapy). Secondly, it was a single-center study with a relatively small sample size (only 239 cases were included). Larger sample clinical analysis is required for further study. Finally, albumin can be affected by non-nutritional factors such as inflammatory state, further studies adjusting factors accounting for inflammatory background (such as C-reactive protein) might probably improve our findings.

## Conclusion

In conclusion, the present study suggests that the GNRI is a simple and effective tool to predict the overall survival time in elderly ESCC patients treated with radiotherapy. Compared with the non-nutritional risk group, the risk of death had increased by 68.8% for moderate risk group and 169.9% for high risk group, respectively.

## Supporting Information

S1 File Excel-Files containing the underlying data. (XLSX)
